# Fast Hand Movements Unveil Multifractal Roots of Adaptation in the Visuomotor Cognitive System

**DOI:** 10.3389/fphys.2021.713076

**Published:** 2021-07-20

**Authors:** Yvan Pratviel, Veronique Deschodt-Arsac, Florian Larrue, Laurent M. Arsac

**Affiliations:** ^1^Laboratoire IMS, CNRS, UMR 5218, Université de Bordeaux, Bordeaux, France; ^2^CATIE, Centre Aquitain des Technologies de l’Information et Electroniques, Talence, France

**Keywords:** multifractality, virtual reality, movement, motor control, nonlinear, variability

## Abstract

Beyond apparent simplicity, visuomotor dexterity actually requires the coordination of multiple interactions across a complex system that links the brain, the body and the environment. Recent research suggests that a better understanding of how perceptive, cognitive and motor activities cohere to form executive control could be gained from multifractal formalisms applied to movement behavior. Rather than a central executive “talking” to encapsuled components, the multifractal intuition suggests that eye-hand coordination arises from multiplicative cascade dynamics across temporal scales of activity within the whole system, which is reflected in movement time series. Here we examined hand movements of sport students performing a visuomotor task in virtual reality (VR). The task involved hitting spatially arranged targets that lit up on a virtual board under critical time pressure. Three conditions were compared where the visual search field changed: whole board (Standard), half-board lower view field (LVF) and upper view field (UVF). Densely sampled (90 Hz) time series of hand motions captured by VR controllers were analyzed by a focus-based multifractal detrended fluctuation analysis (DFA). Multiplicative rather than additive interactions across temporal scales were evidenced by testing comparatively phase-randomized surrogates of experimental series, which confirmed nonlinear processes. As main results, it was demonstrated that: (i) the degree of multifractality in hand motion behavior was minimal in LVF, a familiar visual search field where subjects correlatively reached their best visuomotor response times (RTs); (ii) multifractality increased in the less familiar UVF, but interestingly only for the non-dominant hand; and (iii) multifractality increased further in Standard, for both hands indifferently; in Standard, the maximal expansion of the visual search field imposed the highest demand as evidenced by the worst visuomotor RTs. Our observations advocate for visuomotor dexterity best described by multiplicative cascades dynamics and a system-wide distributed control rather than a central executive. More importantly, multifractal metrics obtained from hand movements behavior, beyond the confines of the brain, offer a window on the fine organization of control architecture, with high sensitivity to hand-related control behavior under specific constraints. Appealing applications may be found in movement learning/rehabilitation, e.g., in hemineglect people, stroke patients, maturing children or athletes.

## Introduction

### Nonlinear Movement Coordination

Most activities of daily human life depend on adequate physical interactions between the individual and its environment. This way, the capacity to reach a target obviously represents a critical function of the movement system repertoire. Beyond apparent simplicity, the timely movement of a hand from one target to another has intricate roots in perceptual, motor and cognitive instances. Successful visuomotor behavior requires coordinated activities of these instances that cohere to form an adequate executive. Although most of sensorimotor and cognitive systems have been extensively described through their independent components, explanations remain more elusive when it comes to consider their proper functioning as a whole, which, moreover, makes the essential phenomenon of adaptation difficult to grasp (Torre et al., 2019). To overcome this difficulty, a great deal of attention has been paid in recent years to the main characteristic of interdependencies within and between component activity in complex systems, governed by nonlinear processes, and spanning multiple and nested temporal and spatial scales. Paying specific attention to the interaction between system components rather than to the component activities themselves is thought to better describe the emergence of new cognitive structures (Dixon et al., 2012; Anastas et al., 2014; Wijnants, 2014), wherein the *ad hoc* capacity of the whole system to apprehend new rules dictated by a changing environment depends on interactions between scales. The multifractal formalism of human behavior has been a reliable and popular approach to focus on nonlinear interactions that shape adaptive system flexibility (Ihlen and Vereijken, 2013; Carver et al., 2017; Bell et al., 2019; Torre et al., 2019).

Researchers have notably explored (multi)fractal cognitive coordination of sensorimotor systems beyond the confines of the brain by exploring motor time series (Wijnants, 2014). This approach assumes that temporal and spatial details of movement dynamics are a rich source of information about the hierarchical organization of the whole movement system linking the brain, the body and the environment. On this account, the aggregation of sequential response times (RTs) may reflect the dynamic organization of multiplicative interactions that span a number of nested hierarchical and temporal scales, which can be reliably quantified by analyzing the multifractal structure in the temporal dynamics of the system output.

### The Multifractal Formalism

Multifractality provides a fine analysis to refer to the sort of structural nesting in a system output time series. The multifractal formalism is an extension of the concept of fractality defined by Mandelbrot as “a shape made of parts similar to the whole” (B. Mandelbrot in Feder, 2013). When zooming on a fractal, one can observe that the structure at finer scales contains nested versions of the very same structure observed at coarser scales. What the multifractal approach offers in comparison to monofractal analyses is that nesting patterns can vary both within and across scales. Thus, multifractality has been described as a finer analysis of the statistical structure in time series (Delignieres and Marmelat, 2012; Delignières and Marmelat, 2013) which adds significant value to the exploration of the adaptive flexibility of the system organization when adopting new operating rules (Anastas et al., 2014). In the output signal, multifractality occurs in the distribution of variance or any metrics of fluctuation amplitude over multiple observational scales (Kantelhardt et al., 2002; Ihlen and Vereijken, 2013; Kelty-Stephen et al., 2013). The scale-dependent fluctuation, which is the root of multifractality, can be estimated by a number of methods, each with different properties that make some of them more suitable for particular types of signal (Eke et al., 2012; Mukli et al., 2015). In cognitive and movement science, signals fluctuations as a function of observational scales have been estimated through computations of the standard deviation in the signal summation and conversion (SSC) analysis (Eke et al., 2000), through wavelet transform best described by the wavelet leader (WL) approach (Jaffard, 2004; Jaffard et al., 2007), and through a residual root mean square error after detrending time series with a set of observational windows, performed with the detrended fluctuation analysis (DFA) (Peng et al., 1994).

The multifractal formalism is nicely described in inspiring articles (e.g., Kantelhardt et al., 2002; Ihlen and Vereijken, 2010, 2013; Kelty-Stephen et al., 2013; Mukli et al., 2015). In essence, current hypotheses stem that multiplicative interactions in the organism involve fractal temporal correlations in output time series, and, more saliently, the presence of a range of fractal correlations within the same time series, which is referred to as “multifractality.” Where the monofractal exponent represents the scaling behavior in a system, the width of the spectrum of co-existing exponents provides the degree of system multifractality (Kantelhardt et al., 2002; Eke et al., 2012; Ihlen and Vereijken, 2013). Critical steps have been taken in anchoring a multifractal approach in the study of nonlinear processes that govern the complex movement system (Ihlen and Vereijken, 2013; Kelty-Stephen et al., 2013, 2016; Anastas et al., 2014; Bell et al., 2019; Mangalam and Kelty-Stephen, 2020), thus providing to date a reliable window to explore proper system behavior and its adaptative capacity to an ever-changing environment.

### Multifractality and *ad hoc* Adaptation

After an era of accumulating evidence that behaviors in young and healthy movement systems exhibit fractal characteristics degraded with disease and aging, finer analyses of motor time series have increasingly exploited the roots of multifractal formalisms. These analyses have widened the scope of system functioning exploration, noticeably toward *ad hoc* adaptation reflected in nonlinear dynamics (Anastas et al., 2014; Likens et al., 2017; Torre et al., 2019). How multifractal metrics are able to probe system adaptation is a key – and not trivial – question that has been nicely addressed in a fairly comprehensive manner in a recent work (Torre et al., 2019). In an experiment comparing the gradual deprivation of sensory inputs in healthy participants to a deafferented man as a pathological-limit case, the authors clarified the role of fractal properties in sensorimotor dynamics. Multifractality demonstrated high sensitivity to effective (*ad hoc*) adaptation imposed by experimental constraints (sensory deprivation). Moreover, it was possible to distinguish this adaptation from adaptability, better reflected in the monofractal behavior of the movement system. Each concept can be explored independently thanks to an extensive approach of movement coordination (Arsac, 2021).

### Hand Movement Behavior

By providing consistent results with the above intuitions, some recent research can help illustrate how multifractal analysis is applied, and what kind of hand behavior has been associated with multifractality. Nonaka and Bril (2014) studied the manufacture of stone and glass beads, analyzing the movement of the craftsman’s hand on a hammer. More skilled craftsmen demonstrated greater multifractality. Likens et al. (2017) observed participants writing an essay by typing it on a computer keyboard. Pairs of raters evaluated the essays based on holistic quality and analytic subscales (introduction, organization, grammar, cohesion, structure…). Greater quality essays were associated with broader multifractal spectra obtained from keystroke interval time series. Tracking hand movements during a standard task of card-sorting, Anastas et al. (2014) associated multifractality in hand trajectories to the underlying adaptive flexibility in executive function. Preschooler participants were asked to sort cards according to an unknown rule, which they had to guess based on feedback about correct card placement. The authors observed a multifractal behavior in densely sampled hand movements, with clear nonlinear origins demonstrated by phase-randomized surrogate data testing, both in the beginning of the sorting task and in the second half, after the rule changed and the participants had to induce it once again. More recently, by combining a multifractal approach of hand movements with the standard Fitts task and speed-accuracy trade-off as an experimental attempt, Bell et al. (2019) showed that nonlinear amplification of movement variability through interaction across scales supports greater accuracy in manual aiming.

### The Focus on Short-Range Dynamics

It is not trivial to observe that in above-described experiments, fine grained fluctuations in hand trajectory have been captured using densely sampled hand movements, not intervals duration between successive (rhythmic) events. The characteristics of the multifractal behavior were thus explored in very short temporal scales. The underlying hypothesis is that the cascading organization of movement coordination reflects a multifractal tensegrity in which nonlinear interactions across scales are reliably reflected in the short-scale multifractal analysis. As a possible application, short-scale multifractal analysis makes sense for exploring adaptation reflected in fast sequences of hand movements under different task settings. Exploiting the Fitts task as an experimental attempt, a task in which performance under critical time pressure is an essential foundation, it has been shown that global constraints can produce changes in the fine-scale dynamics of the hand trajectory (Wijnants et al., 2012), interpretatively captured by a short-scale multifractal analysis (Bell et al., 2019). The main intuition is that a fractally scaled measurement inherently exhibits a scale-invariant decay of variability, so that short-scale behaviors demonstrate close correlation with the longer-range or more global constraints of a task (Palatinus et al., 2013; Anastas et al., 2014; Mangalam et al., 2020a,b,c; Kelty-Stephen et al., 2021). Thus, the multiple fractal results obtained from the analysis of densely sampled movements provide insight into the hierarchy of cross-scale interactions of the movement system.

### A Visuomotor Task Involving Fast Movements of the Hands

Assuming sufficient reliability in above-described short-range multifractal behaviors to infer nonlinear coordination in sensorimotor cognitive control, a focus through the lens of multifractality on the visuomotor system operating in essential interactions like grasping objects or touching targets makes sense. Fast processing in eye-hand coordination is essential for our capacity to quickly reach an object and represents therefore a critical function of the movement system repertoire. Typically, as described above, a wide system that links the brain, the body and the environment must operate efficiently through the nonlinear processes that govern the hierarchical movement coordination.

In an attempt to experimentally explore the organization of the visuomotor system, we use a task that has been designed to assess, or to improve through practice, visuomotor dexterity. This task, called Dynavision (Klavora et al., 1994), involves goal-directed rapid movements of the hands in response to visual stimuli occurring in more or less extended visual search fields. Specifically, targets/buttons that are spatially organized on a vertical board must be hit as quick as possible when they light up in a random order. A replication of the task was recently implemented in virtual reality (VR) (Pratviel et al., 2021), with the significant advantage that the VR setup includes tracked VR controllers held in the participant’s hands, which allows hand movements to be captured with sufficiently fine-grained measurements.

### Manipulating the Visual Search Field to Set a Global Task-Constraint

The visuomotor task in VR (VMVR) offers a great flexibility to study visuomotor dexterous behavior under varying visual input constraints (Pratviel et al., 2021). For the participant, a successful execution relies on adequate coordination between visual search, the voluntary eye movements that actively scan the environment to capture task-relevant information, and cognitive control in movement system. Searching relevant information in a specific field of view represents *per se* a global constraint to which the entire movement system must adapt. As a first reasonable intuition, an extended visual search field should exacerbate the need of fine perceptual-motor coordination through cognitive adaptation; by contrast, the task constraint is relaxed when the search field is reduced. An appealing observation in previous works has been that lower view field (LVF) movement times are consistently lower than upper view field (UVF) ones (Stone et al., 2019). This LFV advantage might have its roots in evolutionary pressure selecting for feeding and foraging behavior (Milner and Goodale, 2006), whose retinal (Curcio and Allen, 1990) and neural correlates explored by fMRI (Rossit et al., 2013; Maltempo et al., 2021) have been identified.

### Hypotheses

In the present study, we used the VMVR task as an experimental attempt to describe changes in eye-hand cognitive coordination that is supposed to arise from multiplicative cascade dynamics across temporal scales that link the brain, the body and the environment. We plant the flag of short-scale multifractality as a suitable approach to extract characteristics of system-wide *ad hoc* adaptations. Fine-grained dynamics in hand movements were extracted from densely sampled VR controller displacements under manipulated visual contexts.

We hypothesized that specific but subtle adaptations in the complex cognitive structure that emerges in LVF and UVF experimental situations are reflected in the multifractal behavior of each hand. A more marked adaptation, reflected in a wide multifractal spectrum of hand dynamics is expected when the visuomotor task takes place in a large visual search field.

## Materials and Methods

### Apparatus

**FIGURE 1:**
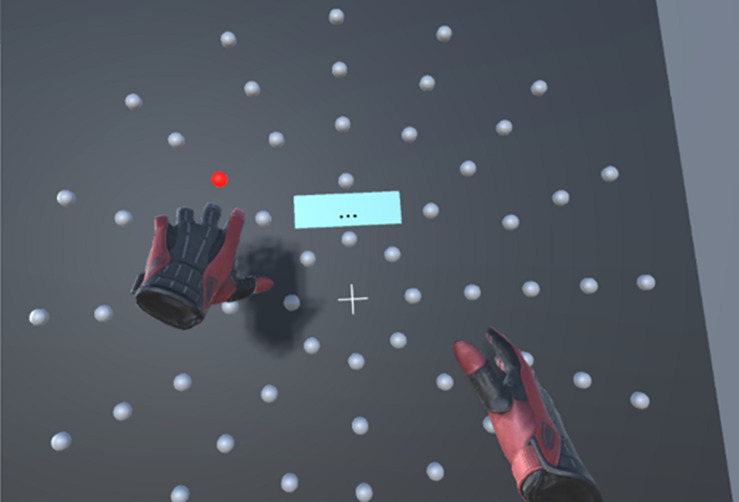
View of the display in virtual reality (VR). The red light represents the active target to be hit. Gloves represents the controllers the user holds in his hands.

The experiment took place in a virtual environment (Figure 1), developed by the CATIE (Centre Aquitain des Technologies de l’Information et Electroniques, Bordeaux, France) with the Unity software (Unity Technologies, San Francisco, CA, United States), and delivered via an HTC Vive Pro headset with two manual controllers (HTC America, Inc., Seattle, WA, United States). The position of each controller in the VR environment was displayed as virtual gloves, thus constituting a visual feedback.

The VMVR – derived from the Dynavision (D2; Dynavision International LLC, West Chester, OH) – consists in hitting as quickly as possible targets/buttons spatially arranged around concentric circles on a vertical board (120 cm × 120 cm). Buttons are successively lit up after being reached by the hand-held VR controllers. More details can be found in Pratviel et al. (2021).

### Participants

A total of 64 healthy sport sciences students gave their informed consent to participate to this program, that was part of their academic curriculum and for which they received credits. The institutional review board (Faculte des STAPS Institutional Review Board Univ. Bordeaux) approved the procedure that respected all ethical recommendations and followed the declaration of Helsinki. All the participants had normal or corrected-to-normal vision. None of them had any prior experience with the present task. Participants were instructed to not consume caffeine or alcohol at least 24 h before the experiment.

Two participants’ data were corrupted, and three others were identified as outliers in the multifractal analysis. Therefore, data are processed for 59 participants [32 females, 27 males, 19 ± 1.8 years (range 18–25), 171.1 ± 9.5 cm, 63.3 ± 9.5 kg, five of them are left-handed].

### Procedure

The experiments were conducted with three gradually challenging modes, imposed by the characteristics of the visual search field. “LVF” and “UVF” mean that only upper-half and lower-half buttons, respectively could light up. The LVF advantage has been stressed in introduction. The mode called “Standard” mean that only lower-half and upper-half buttons, respectively, could light up.

Prior to their arrival, participants completed a questionnaire with socio-demographic information. Their hand preference was assessed with a Modified Edinburgh Handedness Questionnaire (Oldfield, 1971; Cohen, 2008).

Participants discovered the apparatus for the first time. In agreement with previous recommendations for familiarization (Wells et al., 2014), prior to executing the VMVR task in different modes, each participant ran two one-minute tests in Standard mode. During these pre-tests, participants were instructed to find a comfortable distance away from the virtual board so that they could reach and see all the targets. The height of the virtual board display was adjusted so that the participant’s eyes faced the small LCD screen, just above the center of the display (see Figure 1). Participants were instructed to preferentially switch-off the buttons on the left with the left controller, and conversely, but it was not mandatory. Then, they passed each condition (LVF, UVF, and Standard) two-times (test-retest) in a random order, each test lasting 60 s.

### Acquisition of Hand Movements

In each condition, hand movements were acquired from the HTC Vive controllers’ displacements using the SteamVR plugin. Instantaneous controllers’ positions were sampled at 90 Hz with a spatial resolution of 1 mm. The hand movement time series for each hand (upon which the multifractal analysis has been performed, see below), were calculated by the euclidean distance between each pair of consecutive points (thus combining the x, y, and z axis). Based on the results from the Modified Edinburgh Handedness Questionnaire, data from left and right hands were properly labeled as coming from dominant and non-dominant hands. Data analysis was performed using Matlab (Matlab 2019b, Matworks, Natick, MA, United States).

### Signal Pre-processing

**FIGURE 2:**
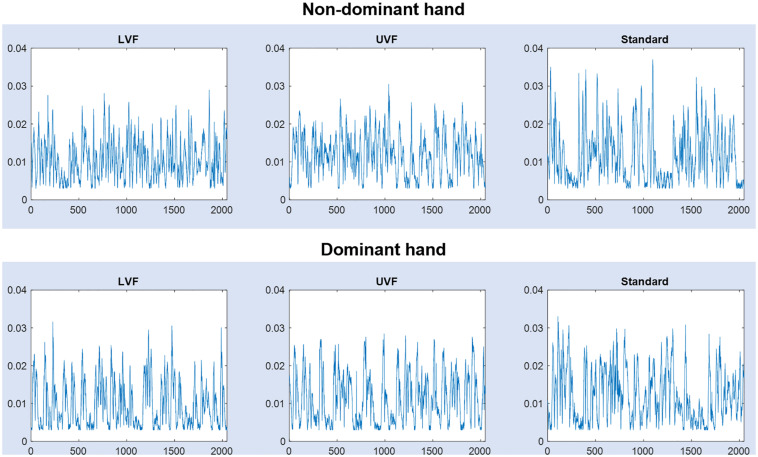
Hand movement signals (interpoint distance in meters vs. time) from one subject, in the three conditions [lower view field (LVF), upper view Field (UVF) and Standard], for the dominant and non-dominant hands.

Before further analyses of hand movement time series, every signal sample that indicated a displacement below the spatial resolution (3 mm for 3D hand-displacements) was deleted. Then, we kept the last 2048 samples for each condition, in each subject. Representative signals are shown in each condition LVF, UVF, Standard and for each hand (dominant and non-dominant) in Figure 2.

### Multifractality in Hand Displacement Time Series

The multifractal characteristics of 3D hand-displacement time series were estimated by using a focus-based multifractal analysis based on a DFA (Mukli et al., 2015). This method is the multifractal adaptation of the DFA developed by Peng et al. (1994), and estimates fractal properties over a range of moment orders *q*. The main principle is that negative *q* values magnify small fluctuations in the series, while positive *q* values magnify large fluctuations, making it possible to obtain a range of scaling exponents that finely describe fractal characteristics.

#### Multifractal Detrended Fluctuation Analysis

Given an initial signal x of size L, the DFA algorithm follows the following steps:

(1) We computed the cumulated sum from which the mean is subtracted:

(1) $$y\left(i\right)=\sum_{k=1}^{i}\left[x_{k}-\left\langle x\right\rangle \right],i=1,\ldots ,L$$

(2) Then *y*(*i*) is divided into *N_s_* = floor(L/s) nonoverlapping boxes of length s. The scales s were constructed equidistantly on a logarithmic scale. For each box ν, a local trend *y*_ν_ was calculated by a least-square approximation.
(3) The variance F^2^(ν,*s*)of the detrended time-series was then calculated for each box ν and scale *s*:

(2) $${\text{F}}^{2}\left(\nu ,s\right)=\frac{1}{s}\int_{i=1}^{s}{\left\{y\left[\left(\nu -1\right)s+i\right]-y_{\nu }\left(i\right)\right\}}^{2}$$

(4) The next step consisted in calculating the *q*th order fluctuation function by averaging the variance F^2^(ν,*s*) over all the *N_s_* boxes.

(3) $${\text{F}}_{q}\left(s\right)={\left\{\frac{1}{N_{s}}\int_{\nu =1}^{N_{s}}{\left[{\text{F}}^{2}\left(\nu ,s\right)\right]}^{\frac{q}{2}}\right\}}^{1/q}forq\neq 0$$

(4) $${\text{F}}_{q}\left(s\right)=\text{exp}\left\{\frac{1}{2N_{s}}\int_{\nu =1}^{N_{s}}ln\left[{\text{F}}^{2}\left(\nu ,s\right)\right]\right\}forq=0$$

The case *q* = 2 supposedly corresponds to the monofractal Hurst exponent calculation, although the use of the focus-based approach might provide a shifted *H(2)* value (see Figure 4 in Mukli et al., 2015, even on synthetical signals). Equation 3 shows that the statistical moment *q* acts as a filter emphasizing small and large fluctuations for *q* < 0 and *q* > 0, respectively.

(5) At last, the fluctuation functions F_*q*_(*s*) were logarithmically plotted against the scales *s* for each value of *q*. If the original signal x shows fractal scaling properties, the fluctuation function follows a power law for increasing scales s:

(5) $${\text{F}}_{q}\left(s\right)\propto s^{H\left(q\right)}$$

The generalized Hurst exponent *H*(*q*) yields the multifractal signature of hand movement time series.

#### Focus-Based Multifractal Formalism

In order to calculate *H(q)* with a more robust and unbiased method, we use a reference point (focus) during the regression of fluctuation functions (Mukli et al., 2015). Briefly, this method is based on the fact that, for a signal with finite length, all *q*th order fluctuation functions converge towards an identic point when the signal length L is used as the scale s. Mainly, it prevents the multifractal analysis of empirical time series from being corrupted.

The theoretical focus point *S(q,L)* has an effect on the statistical errors measured for the regression of the scaling functions. Indeed, forcing the multifractal formalism helps getting a fan-like geometry for signals of finite size, but weakens the correlation coefficient in the estimation of individual Hurst exponents. Even though the correlation coefficient *R*^2^ is sometimes used as a relevant indicator to choose the range of statistical moments *q* to be included in the multifractal analysis, this is less of a concern when using the focus-based analysis; here, the objective is not to run a monofractal analysis on a set of exponents *q*, but rather to use the properties of multifractal signals to get a more reliable estimation of *H(q)* for empirical time series of finite length.

#### Application to Hand Movement Time Series

**FIGURE 3:**
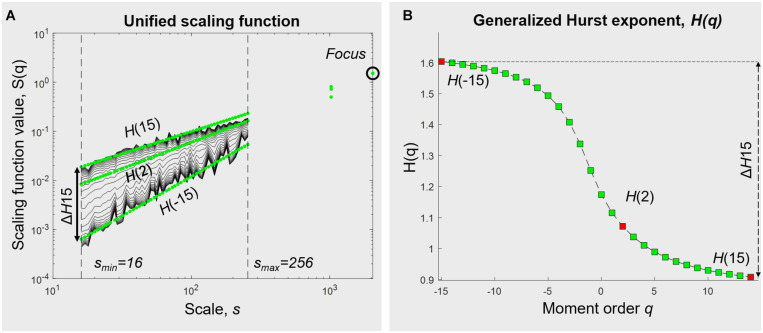
Multifractal analysis of hand movement time series (in this example, in UVF with the dominant hand). **(A)** Unified scaling function from a focus-based multifractal DFA analysis. **(B)** Generalized Hurst exponent, obtained from linear regression over statistical moments *q*. In some extent, *H(2)* represents the monofractal exponent (see Section “Multifractality in Hand Displacement Time Series”). The degree of multifractality Δ*H15* is calculated as the difference between H(–15) and H(15).

Using focus-based MF-DFA with a range of *q* from −15 to +15 and observational scales going from 16 to 256 samples, we plotted the *q*th order fluctuation functions (Figure 3A). The generalized Hurst exponent was obtained for the whole range of *q*-values (Figure 3B). We chose to use N/8 as our maximum window in the multifractal analysis to avoid the influence of eventual drifts.

The choice of the range of *q* values was guided both by previous experiments with physiological time series, e.g., (Nagy et al., 2017; Racz et al., 2018a,b), and by the sigmoidal pattern in the representation of the Hurst exponents across statistical moments *q*, with asymptotic trends for far negative and far positive values of *q*. This phenomena is nicely described by Grech and Pamuła (2012); the authors propose that those asymptotic limits are already reached for a range of *q* going from −15 to +15.

The degree of multifractality was calculated as the difference between the two most distant Hurst exponents, here H(−15)–H(15), and is subsequently called Δ*H15*. With *q* acting as a magnifying lens on small and large fluctuations, Δ*H15* allows us to assess the extent to which the scale-free components depend on *q*.

As each participant performed two runs in each condition, we further use the mean of the Δ*H15* obtained for test and retest in our analysis. The common endpoint of the multifractal analysis is the singularity spectrum, relating the Hölder exponents α and the fractal dimension *f(α)*. With this representation, the multifractal signature lies in the width of the singularity spectrum, Δα. The generalized Hurst exponent *H(q)* and the Hölder exponent α are linked together according to the following equation :

(6) $$\alpha =H\left(q\right)+qH^{\prime }\left(q\right)$$

Then, the fractal dimension *f(α)* is obtained with:

(7) f(α)=q(α-H(q))+1

(see e.g., Equations 9–16 in Kantelhardt et al., 2002). Therefore, Δ*H15* and Δα give a similar estimation of multifractality.

### Surrogate Data Testing

Before concluding on the Δ*H15* calculated from our time series, we need to ensure that the multifractal scaling observed originates effectively from long-range correlations. Actually, several pitfalls have been identified. Mainly, multifractality could derive from heavy tailed probability distribution of signal values (Ivanov et al., 1999; Kantelhardt et al., 2002), linear autocorrelations, or the finite size of the time series. In order to distinguish true multifractality from “multifractal background noise,” a surrogate data testing was conducted following guidelines (Eke et al., 2012).

To test the presence of true multiplicative processes between time scales rather than linear autocorrelations, the original time series were phase randomized using Iterated Amplitude Adjusted Fourier Transform – IAAFT (Schreiber and Schmitz, 1996). This method preserves the probability density function and the power spectral density, but the phase shuffling destroys long-range correlations.

Here, we generate surrogates (*n* = 40) for each time series, and compare the Δ*H15* from the original signal with the ones obtained from the surrogates. As the phase randomization process preserves only linear phenomenon in the series, we consider the signal to be truly multifractal if the Δ*H15* value of the original signal is significantly higher than Δ*H15* of surrogates.

### Additional Testing

As mentioned before, and proposed by Mukli et al. (2015), Δ*H15* has also been assessed using two alternative methods based on the focus approach: SSC and WL.

As a source of comparison, we also computed multifractality using the Chhabra and Jensen method (Chhabra and Jensen, 1989), and discussed some related issues (range of *q*-values, goodness of fit). Detailed results are provided in Supplementary Material 1.

### Statistical Analysis

Statistical analyses were performed using Matlab and the R software R Core Team (2020).

Normal distribution for visuomotor RT and multifractality (Δ*H15*) was assessed using a Shapiro–Wilk test. As a first step, a two-way ANOVA on Δ*H15* with condition (LVF, UVF, and Standard) and hand (dominant, non-dominant) as independent variables was used to detect possible interactions. As interaction was confirmed, it was not possible to analysis each hand separately. Hence, a one-way repeated measures (six-repetitions) ANOVA analysis with a *post hoc* Tuckey test was used to compare Δ*H15* obtained in each condition and for each hand. Data sphericity was assessed with a Mauchly’s test. The effect size was computed using the ω^2^.

In the case where the normality of the data was not established, a Kruskal–Wallis ANOVA with a *post hoc* Bonferroni was performed. The effect size was then computed using the η^2^.

## Results

### Visuomotor Response Time

**FIGURE 4:**
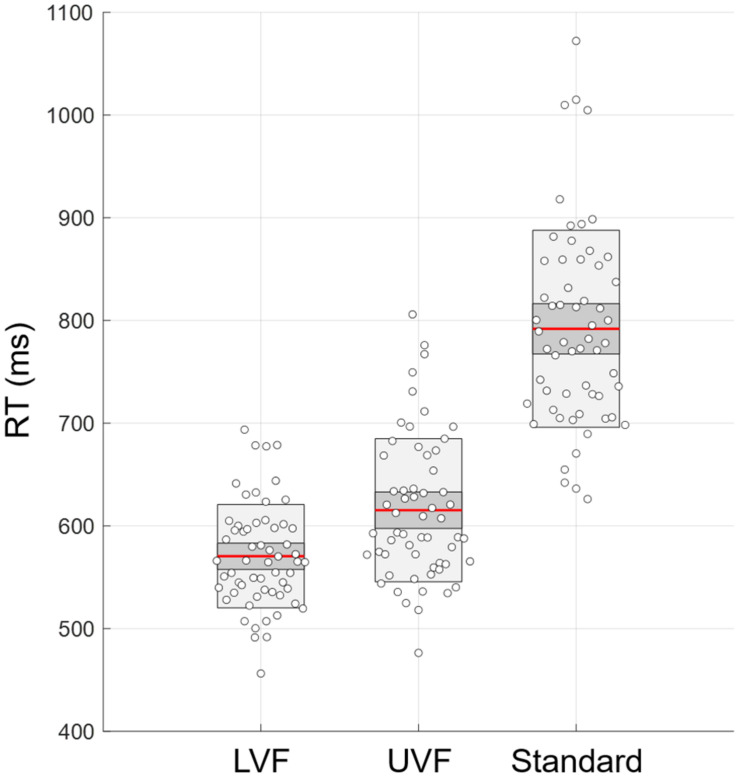
Response times (RT) calculated as the mean of both test and retest RTs for the conditions LVF, UVF, and Standard. Red line, mean of the samples; dark gray, standard deviation; light gray, 95% confidence inteval.

Shapiro–Wilk tests for visuomotor RTs showed the absence of normal distribution in conditions UVF and Standard (*p* < 0.05 for both). A Kruskall-Wallis ANOVA test was used and demonstrated differences across conditions (*Chi*^2^ = 109.8, *p* = 1 × 10^–24^, η^2^ = 0.62). *Post hoc* Bonferroni tests indicated visuomotor RT increasing with task difficulty: RT LVF < RT UVF < RT Standard (all *p* < 2 × 10^–5^). Results for the mean RT of test and retest across the three conditions are displayed in Figure 4.

### Testing for True Multifractality

Δ*H15* were calculated for each surrogate series obtained from phase randomization (IAAFT). Overall, 81% (571/708) of the original series had Δ*H15* significantly larger than their surrogates (78% for the dominant hand series, and 84% for the non-dominant ones). Regarding conditions, in LVF, UVF, and Standard, 81, 88 and 73% of series had Δ*H15* significantly larger than their surrogates, respectively. Subsequent analyses were conducted assuming true multifractal behavior in hand movements, reliably captured by Δ*H15*.

### Multifractal Properties – Δ*H15*

Multifractal behaviors have been obtained from Δ*H15* values (see section “Multifractality in Hand Displacement Time Series”, and a typical example in Figure 3).

**FIGURE 5:**
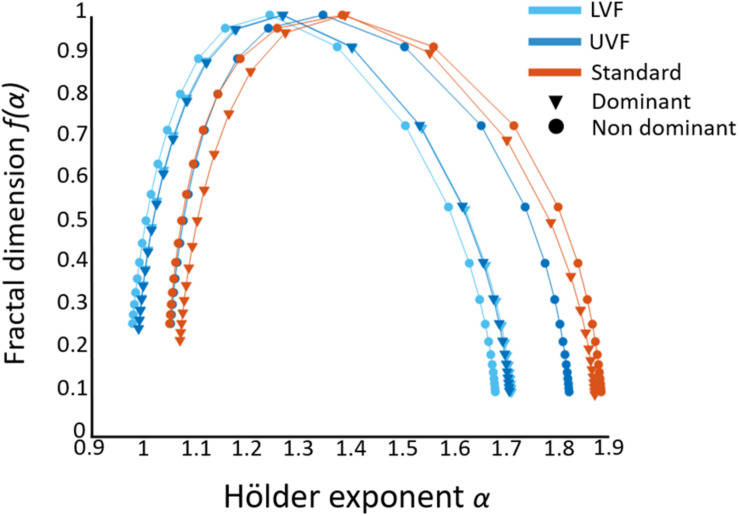
Average singularity spectra obtained from FMF–DFA in the three conditions (distinguished by colors) for both hands (distinguished by markers). The width of the spectra gives a similar information as Δ*H15* to infer the multifractal behavior. In some extent, the position of the singularity spectra along the x-axis gives a clue about the monofractal behavior (strictly speaking, *H(2)*, see Section “Multifractality in Hand Displacement Time Series”). It is worth noting that the focus-based method, used to enforce the multifractal formalism and avoid corrupted spectra, might influence alpha values, and specifically *H(2)* as an indicator of monofractality. Although mean *H(2)* (1 < *H(2)* < 1.2) seems to indicate somewhat persistent behavior, this is a consequence of applying a focus-based approach on our data (see Section “Multifractality in Hand Displacement Time Series”).

To provide full details about the multifractal analysis, average singularity spectra (representing *f(α)* vs. α, see section “Multifractality in Hand Displacement Time Series”) are provided in Figure 5.

Regarding Δ*H15* analyses, Shapiro–Wilk tests indicated normal distribution of the data in each condition (LVF, UVF, and Standard) for both hands (dominant and non-dominant) (all *p* > 0.21).

**FIGURE 6:**
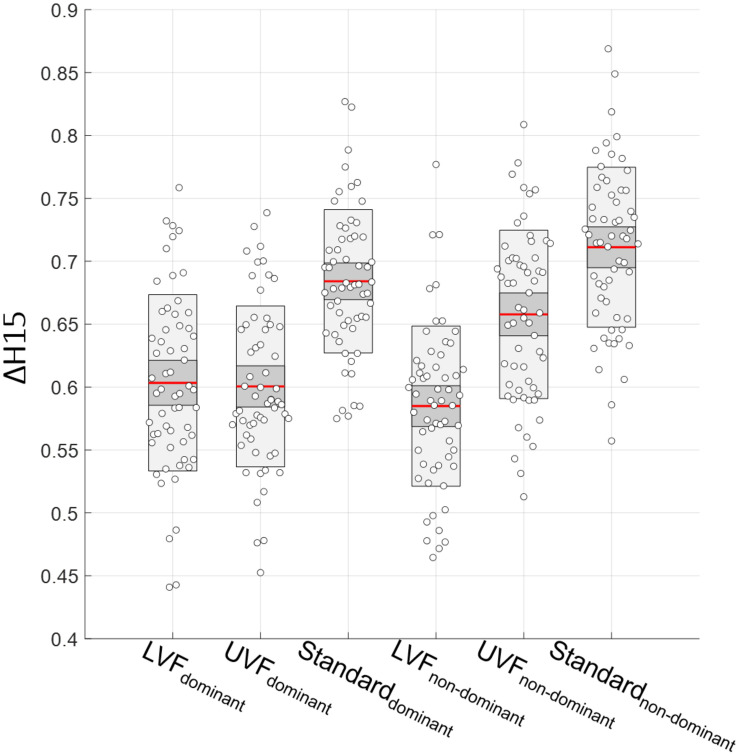
Degree of multifractality Δ*H15* in the three conditions (LVF, UVF, and Standard) for the dominant and non-dominant hands (from left to right).

As mentioned before, we used the mean of Δ*H15* from test and retest in our multifractal analysis. First, a paired *t*-test showed no differences in Δ*H15* between test and retest in each condition, for each hand (all *p* > 0.23). Then, one-way ANOVAs with repeated measures on both the test [*F*(5,353) = 21.67, *p* = 7 × 10^–19^] and retest [*F*(5,353) = 28.11, *p* = 6 × 10^–24^] data led to similar results. Therefore, we confirmed our choice to use the mean of test and retest Δ*H15* values for our analysis. The results of the multifractal analysis are shown in Figure 6.

A two-way ANOVA performed with condition and hand as independent variables showed an interaction effect [*F*(2,353) = 10.37, *p* = 4.22 × 10^–5^]. The ANOVA with repeated measurements highlighted differences between Δ*H15* measured on each condition for both hands [*F*(5,353) = 39.84, *p* = 6 × 10^–31^, ω^2^ = 0.34]. Mauchly’s Test of Sphericity indicated that the assumption of sphericity was not violated (*W* = 0.745, *p* = 0.285). *Post hoc* Tuckey tests indicated higher Δ*H15* values for the Standard condition compared the LVF and UVF ones in both hands (all *p* < 1 × 10^–4^). Moreover, we found no differences between LVF and UVF for the dominant hand (*p* = 1.00), whereas there is one for the non-dominant hand (*p* = 3.14 × 10^–8^). All the results from *post hoc* Tuckey are presented in Table 1.

**TABLE 1:** Results from *post hoc* Tuckey for the degree of multifractality Δ*H15*.

Condition 1	Condition 2	Difference of means	*p*-value
LVF dominant	UVF dominant	0.00	1.00
LVF dominant	Standard dominant	–0.06	2.08 × 10^–8^
LVF dominant	LVF non-dominant	0.02	0.62
LVF dominant	UVF non-dominant	–0.05	6.28 × 10^–5^
LVF dominant	Standard non-dominant	–0.11	2.07 × 10^–8^
UVF dominant	Standard dominant	–0.08	2.07 × 10^–8^
UVF dominant	LVF non-dominant	0.02	0.77
UVF dominant	UVF non-dominant	–0.06	1.96 × 10^–5^
UVF dominant	Standard non-dominant	–0.11	2.07 × 10^–8^
Standard dominant	LVF non-dominant	0.10	2.07 × 10^–8^
Standard dominant	UVF non-dominant	0.03	0.23
Standard dominant	Standard non-dominant	–0.03	0.20
LVF non-dominant	UVF non-dominant	–0.08	3.14 × 10^–8^
LVF non-dominant	Standard non-dominant	–0.13	2.07 × 10^–8^
UVF non-dominant	Standard non-dominant	–0.05	9.64 × 10^–5^

## Discussion

The present study provides additional support to the intuition that multiplicative cascade dynamics may govern nonlinear interactions across scales in the hierarchical organization of movement coordination, through three main original findings : (i) densely sampled hand movements during a visuomotor task exhibit multifractal dynamics; (ii) the degree of multifractality varied with task constraint imposed by manipulating the visual search field; (iii) multifractal-based metrics are sensitive enough to highlight subtle differences in behavior of the dominant vs. the non-dominant hand.

These results add significant support to the notion of body-wide coordinated fluctuations as a process developing through interactions across multiple scales (Mangalam and Kelty-Stephen, 2020). They plead for the virtue of analyzing non-linear interactions estimated by multifractal geometry as a sign of effective *ad hoc* adaptation (Torre et al., 2019). An adaptive behavior might exploit a larger spectrum of fractal dynamics, which enhances both motor and perceptual mechanisms (Mangalam et al., 2020c). Taken together, these observations could form the focus of future follow-up strategies for improving visuomotor dexterity through motor learning, or restoring it through rehabilitation, wherein VR might bring significant value.

### Multifractality in Hand Movements Unveils Interactions Across Scales in the Cognitive System, Supporting Effective Adaptation

Most eye-hand coordinated activities in humans take place in the LVF (Previc, 1990), which has structured a so called LVF advantage over years (Danckert and Goodale, 2001, 2003; Khan and Lawrence, 2005; Krigolson and Heath, 2006; Rossit et al., 2013; Stone et al., 2019). This allowed us to postulate that our VR situation imposing visual search in the lower part of the virtual board (LVF) represents the least constrained visuomotor activity in our conditions. By demonstrating >80% nonlinear dynamics with our surrogate data test, the present study reinforces the intuition that hand movements are not additively decomposable. As a direct consequence, insights from the multifractal formalism that is grounded in multiplicative cascading (not additive) interactions can be thought to provide a well-suited tool to decipher a hierarchical coordination across scales in the operating cognitive system, based on perceptual-motor fluctuations (Van Orden et al., 2005; Diniz et al., 2011; Ihlen and Vereijken, 2013; Wijnants, 2014). On this account, the change in multifractal hand behavior reported here (Figure 6) when the visuomotor task was performed with a larger visual search field (LVF and UVF vs. Standard) is in agreement with our first hypothesis that the degree of multifractality in hand movements reflects the adaptative capacity of a widely coordinated sensorimotor system.

Although effective adaptation has been studied in parallel with adaptability, by exploring comparatively multifractal and monofractal behaviors (Torre et al., 2019), one can hardly infer monofractal behavior in the present study, that focused on multiplicative cascade patterns (Figure 2) analyzed through the multifractal formalism (Kelty-Stephen and Wallot, 2017) using a focus-based method (Mukli et al., 2015). Alternative methods seems to be better suited to infer monofractal behaviors in time series (Roume et al., 2019).

The larger multifractal spectrum width (Δ*H15*) that we observed here in Standard, when compared to LVF and UVF, is in agreement with an increased multifractality in a sensorimotor task when degrading sensory information (Torre et al., 2019). In the same vein, the increased Δ*H15* here is in line with nonlinear amplification (multifractal spectrum width) of hand movement variability that supports greater accuracy in manual aiming during the Fitts task (Bell et al., 2019). By extension, one can conclude that the healthy adaptation of cognitive-motor performance to several sources of external variation is essentially reflected in a rising degree of movement multifractality, a view fully consistent with an increased repertoire of interactivity that supports an adequate response to changes in environment. As underlined in Torre et al. (2019), the association between enhanced multifractality and effective *ad hoc* adaptation observation may not hold in all contexts. Patients or elderly subjects could demonstrate a loss of adaptivity that prevents the enrichment of nonlinear interactions in cognitive control when facing constraints, which is reflected in unchanged or even degraded multifractality. One can also find evidence that the (excessive) complexification of cognitive tasks may lead to reduced multifractal spectrum width (Ihlen and Vereijken, 2013), an extreme case that was obviously not reached in our conditions (Figure 6). Interestingly, in the intermediate scenarios, the insights provided by the multifractal formalism on adaptation do not appear as all-or-nothing, as demonstrated by Torre et al. (2019) who highlight progressive changes in multifractality as a function of gradual sensory deprivation, as well as a significant relationship between the two. The capacity of the multifractal approach to reveal subtle, progressive adaptation when adapting to gradual constraints is of critical importance to explore flexibility in visuomotor control, or even any other cognitive architectures associated to control in the movement system (Arsac, 2021). On this account, the present study adds significant value, by revealing that the dominant and non-dominant hand exhibit singular changes in degree of multifractality when adapting to the (intermediate) constraint imposed by visual search in the UVF.

### Multifractality in Dominant vs. Non-dominant Hand in UVF

Despite a Visual search field of the same size, the visuomotor task in UVF differs from LVF due to the LVF advantage inherited from evolutionary processes supporting foraging and feeding behavior (Milner and Goodale, 2006). In agreement, the visuomotor performance quantified here by RTs showed that this experimental situation UVF lies between a “performant” LVF situation and a “difficult” Standard situation (Figure 4). An appealing result here is that a difference in the degree of multifractality, and therefore a difference in visuomotor adaptation, between the dominant and non-dominant hands is observed only in this particular situation UVF. First of all, a gradual change in multifractality in the non-dominant hand, where Δ*H15* reached an intermediate level between the least (LVF) and the more constrained (Standard) tasks confirms that the multifractal formalism offers a reliable glimpse on adaptive capacities linked to interdependencies governed by nonlinear interactions. Second, it is not surprising at first sight that the non-dominant hand would have to adapt more than the dominant hand, which is again reflected in Δ*H15*. At this stage, the question that arises is why Δ*H15* reflects a hand-specific adaptation in UVF, a hand-specificity observed neither in LVF nor in Standard (Figure 6).

Before discussing possible interpretations, one should keep in mind the main objective of the present study: to consider the fact that the multifractal formalism helps decipher specific adaptation between hand use and visual search situations is *per se* an interesting result. Yet, this is where motion-based multifractal approaches have their limitations, as without direct measures of brain connectivity, one can hardly infer the very neural mechanisms underlying adaptation, with the multifractal approach actually focusing on the emergent control architecture. Although changes in network connectivity have been linked to changes in multifractal sensorimotor behavior when adapting to sensory manipulations (Vergotte et al., 2018), the authors explored connectivity in the sensorimotor cortex thanks to near infrared spectroscopy to elaborate their reasoning, an option not available in the present study.

Adopting a coherent line of reasoning, the non-dominant hand would need more adaptation in each “complexified” situation, namely UVF where the analysis of Δ*H15* confirmed this hypothesis, but also Standard where, in contrast, both hands exhibited similar multifractality (Figure 6). An interpretative hypothesis might be found in the facilitating mechanisms of motor performance described by several authors, relative to both the hand and the space in which it acts. First, distinct neural control mechanisms have been identified between the dominant and non-dominant arms (Sainburg and Kalakanis, 2000). Neuroimaging showed greater recruitment of visual and motor regions when using the non-dominant hand, but greater recruitment of motor planning regions for the dominant one (Kirby et al., 2019). One could thus imagine slightly different cognitive architectures linking the brain, the body and the environment when it comes to control the movement of the dominant or the non-dominant hand: with a richer repertoire of interactions elicited by motor planning in the dominant hand, and visual feedback of trajectory corrections in the non-dominant hand. Thus, the reason why hand-specific architectures demonstrate similar degree of multifractality in LVF may rely on mechanisms at the origin of the natural LVF advantage relying on higher density of ganglion cells in the peripheral retina processing the LVF (Curcio and Allen, 1990) and more efficient pre-cortical processing of LVF information. This advantage combined with greater reliance on visual information in non-dominant hand architecture may flatten the difference in needed adaptation between dominant and non-dominant hand specifically in LVF, which could explain similar Δ*H15* observed here in this particular situation. The LVF advantage disappears in the UVF situation, which reveals the greater adaptation in the non-dominant hand reflected in a higher degree of multifractality (Figure 6). As to the Standard condition, which imposes visual search in both fields LVF and UVF, the LVF advantage operates in successful hits of the non-dominant hand, which might explain similar Δ*H15* observed in this situation in the present study (Figure 6). Obviously, the hand specific networked interactions that are hypothesized here need further investigations including direct brain connectivity measurements. At this stage yet, our results confirm previous suggestions that the multifractal formalism offers an interesting glimpse of fine coordination in the movement system behavior.

### Information Lying in Densely Sampled vs. Event Time Series

It has been brought to our attention that analyzing the fractal behavior in inter-hit duration series could make a significant contribution to a thorough fractal-based approach of coordination in movement system. As our experiment was not initially designed to get event-series, each task – lasting only 60 s – provided few successive hits (maximum was 132, 126 and 99 for LVF, UVF, and Standard, respectively). Individual event-series were obtained by stitching test and retest runs – although this is not a recommended procedure to reliably extract a monofractal behavior (Marmelat and Meidinger, 2019) – to provide event-series amounting to a maximum of 260, 248, and 190 samples, respectively. When applied to these series both a DFA-based analysis and the ARFIMA(0,d,0) model (using Whittle approximation of the maximal likelihood estimator) demonstrated scaling exponents around 0.5 (Supplementary Material 2), which is characteristic of white noise and in line with the random nature of the stimulus. To conclude, despite obvious limitations in building event-series as said above, it seems interesting to notice that in our conditions, relevant information did not lie in the event-series (due to the nature of the task), but in the fine-grained features of movement dynamics, as initially hypothesized in this study. This latter point highlights the strength of the densely sampled approach of movement coordination we used here, inspired by recent research providing converging evidence that adaptation to global task constraints can produce changes in finer-scaled movement dynamics (e.g., Bell et al., 2019). Thus, the present work brings additional evidence that an adequate multifractality analysis of movement behavior may shed light on the movement’s system hierarchy of cross-scale interactions.

## Conclusion and Perspective

The present study supports the intuition of interaction-dominance in the system wide cognitive control of visuomotor dexterity. In agreement with previous studies, fast dynamics in hand movement behavior exhibit multifractal properties that offer an anchor to explore adaptations in an emergent cognitive control. Beyond the observation that multiplicative cascade dynamics might represent a fundamental organization of cognitive movement control, multifractal roots showed tight links with adaptation, a critical property that is broadly associated to health.

Here, as an experimental attempt, we used a VMVR that is easy to reproduce, even outside the laboratory, to reach a wide audience. As VR set-ups offer great flexibility to design perceptual-cognitive tasks and, more saliently, have built-in sensors able to capture densely sampled hand trajectories, the combination of multifractal approaches with VR material is promising. It has not escaped our attention that head movements can be captured by the VR set-up as well but we deplored a too low spatial resolution in our conditions to further exploit a coordinated head-hand multifractal behavior. Although this is an interesting perspective, the approach would require additional tools to get sufficient accuracy in head movement variability. In the same vein, finer analyses of the cognitive structure supporting visuomotor dexterity could be gained by introducing variability in gaze fluctuations thanks to eye tracking; this could prefigure next pertinent steps in merging cognition and movement sciences. We anticipate that future work, possibly combining hand, eye and head dynamics, will fruitfully provide means for understanding system wide control, so as to further consolidate the present findings.

## Data Availability Statement

The raw data supporting the conclusions of this article will be made available by the authors, without undue reservation.

## Ethics Statement

The studies involving human participants were reviewed and approved by the Faculte des STAPS Institutional Review Board Univ. Bordeaux. The patients/participants provided their written informed consent to participate in this study.

## Author Contributions

LA and YP conceived the study and wrote the manuscript. LA, FL, and YP designed the study. YP collected the data. LA, YP, and VD-A analyzed the data. All authors approved the final manuscript.

## Conflict of Interest

The authors declare that the research was conducted in the absence of any commercial or financial relationships that could be construed as a potential conflict of interest.
