# Supplementary material for: Fast Hand Movements Unveil Multifractal Roots of Adaptation in the Visuomotor Cognitive System
Source: Front Physiol. 2021 Jul 20;12:713076. doi: 10.3389/fphys.2021.713076 (PMC8330832; doi:10.3389/fphys.2021.713076)
Supplement: Supplementary file 1 [file Table_1.pdf]

Supplementary Material 1

1 Multifractal properties – assessing *ΔH15* with alternative methods

Here, we detail the results obtained for the processing of hand movement data with two alternative methods for obtaining multifractality while respecting the focus-based formalism: SSC (Signal Summation Conversion), and WL (Wavelet Leader).

1.1 Focus-based SSC

The SCC analysis led to the same 3 outliers (detected using the isoutlier function in Matlab). Thus, the analysis is conducted on the same 59 subjects as the one using DFA.

Shapiro–Wilk tests indicated normal distribution of the data in each condition (*LVF*, *UVF*, *Standard*) for both hands (*dominant* and *non-dominant*) [all $p > 0.30$].

A two-way ANOVA performed with *condition* and *hand* as independent variables showed an interaction effect [$F(2,353) = 10.11$, $p = 5.39 \times 10^{-5}$].

The ANOVA with repeated measurements highlighted differences between *ΔH15* measured on each condition for both hands [$F(5,353) = 35.74$, $p = 1.68 \times 10^{-29}$, $\omega^2 = 0.33$].

Mauchly's Test of Sphericity indicated that the assumption of sphericity was not violated [$W = 0.74$, $p = 0.262$].

Post-hoc Tuckey tests indicated higher *ΔH15* values for the *Standard* condition compared to the *LVF* and *UVF* ones in both hands [all $p < 2 \times 10^{-4}$]. Moreover, we found no differences between *LVF* and *UVF* for the dominant hand [$p = 1.00$], whereas there was one for the non-dominant hand [$p = 4.34 \times 10^{-8}$]. All the results from post-hoc Tuckey are presented in table 1.
To summarize, as indicated in Table 1 and figure 1 below, the focus-based multifractal analysis using SSC provided similar results than the focus-based DFA analysis used in the present work.

Condition 1	Condition 2	Difference in means	p-value
LVF dominant	UVF dominant	0.00	1.00
LVF dominant	Standard dominant	-0.08	2.18×10^{-8}
LVF dominant	LVF non dominant	0.02	0.63
LVF dominant	UVF non dominant	-0.05	1.08×10^{-4}
LVF dominant	Standard non dominant	-0.11	2.07×10^{-8}
UVF dominant	Standard dominant	-0.08	2.09×10^{-8}
UVF dominant	LVF non dominant	0.02	0.78
UVF dominant	UVF non dominant	-0.06	3.22×10^{-5}
UVF dominant	Standard non dominant	-0.11	2.07×10^{-8}

Standard dominant	LVF non dominant	0.10	2.07×10^{-8}
Standard dominant	UVF non dominant	0.02	0.33
Standard dominant	Standard non dominant	-0.03	0.16
LVF non dominant	UVF non dominant	-0.07	4.34×10^{-8}
LVF non dominant	Standard non dominant	-0.12	2.07×10^{-8}
UVF non dominant	Standard non dominant	-0.05	1.57×10^{-4}

Table 1 : Results from post-hoc Tuckey for the degree of multifractality *ΔH15* calculated with focus-based SSC

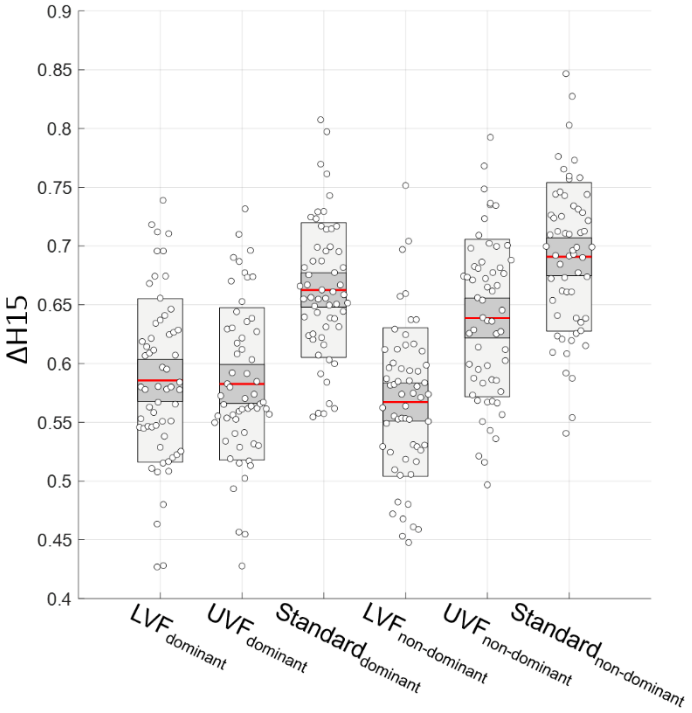

Figure 1 **:** Degree of multifractality *ΔH15* calculated with focus-based SSC in the three conditions (*LVF*, *UVF*, *Standard*) for the dominant and non-dominant hands (from left to right)

1.2 Focus-based WL

The WL analysis led to the 5 outliers (detected using the isoutlier function in Matlab), with 4 of them being different than the ones detected with the DFA and SSC methods. Thus, the analysis is conducted on 57 subjects.

Shapiro–Wilk tests indicated normal distribution of the data in each condition (*LVF*, *UVF*, *Standard*) for both hands (*dominant* and *non-dominant*) [all $p > 0.19$].

A two-way ANOVA performed with *condition* and *hand* as independent variables showed an interaction effect [$F(2,341) = 5.89$, $p = 0.003$].

The ANOVA with repeated measurements highlighted differences between *ΔH15* measured on each condition for both hands [$F(5,341) = 30.97$, $p = 6.64 \times 10^{-26}$, $\omega^2 = 0.13$].

Mauchly's Test of Sphericity indicated that the assumption of sphericity was not violated [$W = 0.653$, $p = 0.059$].

Post-hoc Tuckey tests indicated higher *ΔH15* values for the *Standard* condition compared to the *LVF* and *UVF* ones in both hands [all $p < 5 \times 10^{-6}$]. Moreover, we found no differences between *LVF* and *UVF* for the dominant hand [$p = 1.00$], whereas there was one for the non-dominant hand [$p = 8.53 \times 10^{-5}$]. All the results from post-hoc Tuckey are presented in table 2.

To summarize, as indicated in Table 2 and figure 2 below, with only one exception (*Standard dominant vs. UVF non dominant*) the focus-based multifractal analysis using WL provided similar results than the focus-based DFA analysis used in the present work.

**Condition 1**	**Condition 2**	**Difference in means**	**p-value**
LVF dominant	UVF dominant	0.00	1.00
LVF dominant	Standard dominant	-0.07	2.27×10^{-8}
LVF dominant	LVF non dominant	0.01	0.76
LVF dominant	UVF non dominant	-0.03	0.02
LVF dominant	Standard non dominant	-0.09	2.07×10^{-8}
UVF dominant	Standard dominant	-0.07	2.11×10^{-8}
UVF dominant	LVF non dominant	0.01	0.87
UVF dominant	UVF non dominant	-0.04	8.41×10^{-3}
UVF dominant	Standard non dominant	-0.09	2.07×10^{-8}
Standard dominant	LVF non dominant	0.08	2.07×10^{-8}
Standard dominant	UVF non dominant	0.03	0.02
Standard dominant	Standard non dominant	-0.02	0.42
LVF non dominant	UVF non dominant	-0.05	8.53×10^{-5}
LVF non dominant	Standard non dominant	-0.10	2.07×10^{-8}
UVF non dominant	Standard non dominant	-0.06	5.08×10^{-6}

Table 2 : Results from post-hoc Tuckey for the degree of multifractality *ΔH15* calculated with focus-based WL

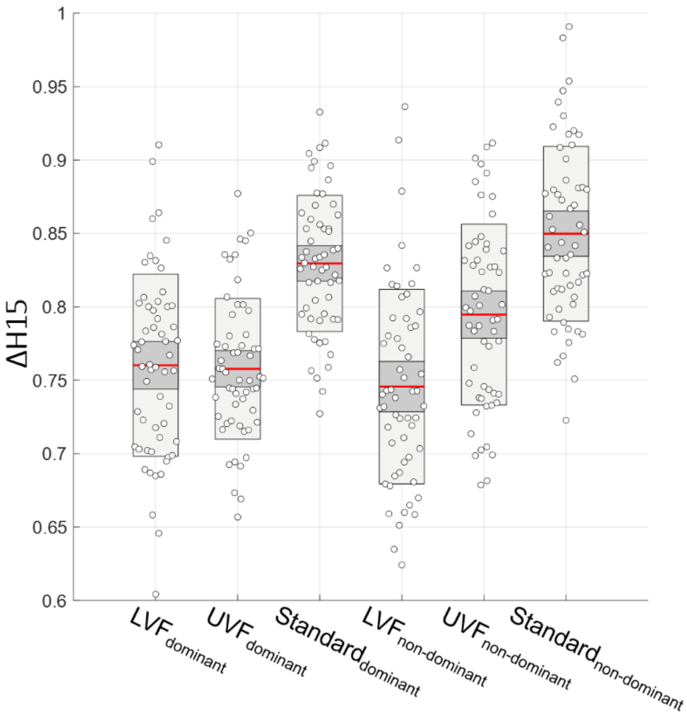

Figure 2 : Degree of multifractality *ΔH15* calculated with focus-based WL in the three conditions (*LVF*, *UVF*, *Standard*) for the dominant and non-dominant hands (from left to right).

2 Fluctuations *vs.* temporal scales goodness of fit

Any multifractal approach of time series requires sufficient precision to establish the linear (log-log) relationship between signal fluctuations and temporal scales. Therefore, the goodness of fit between these variables is not a trivial issue, that may dictate the choice of the range of *q* values allowing a reliable quantification of multifractality when using some methods (see *e.g.* Bell *et al.* 2019). Nevertheless, obtaining high values of R^2 is less prominent with the method used here since it introduces a focus point to get closer to the multifractal formalism (Mukli *et al.* 2015).

To further illustrate this point mentioned in section 2.6 of the manuscript about choosing *q* values and using the focus point method, we present here more figures for the multifractal analysis, including the R^2 calculated for each moment order *q* with and without the use of the focus point (figure 3B below). Although lower R^2 values are obtained using the focus point method, this is not a concern as explained in paragraph 2.6 of the main text. This is the consequence of introducing a focus point, which provides other significant advantages including a formalism closer to multifractality (Mukli *et al.* 2015).

Additional testing in the present work employed the Chhabra & Jensen method to get a multifractal spectrum, which suggests further discussion about the role of goodness of fit in assessing multifractality, as proposed in the next paragraph.

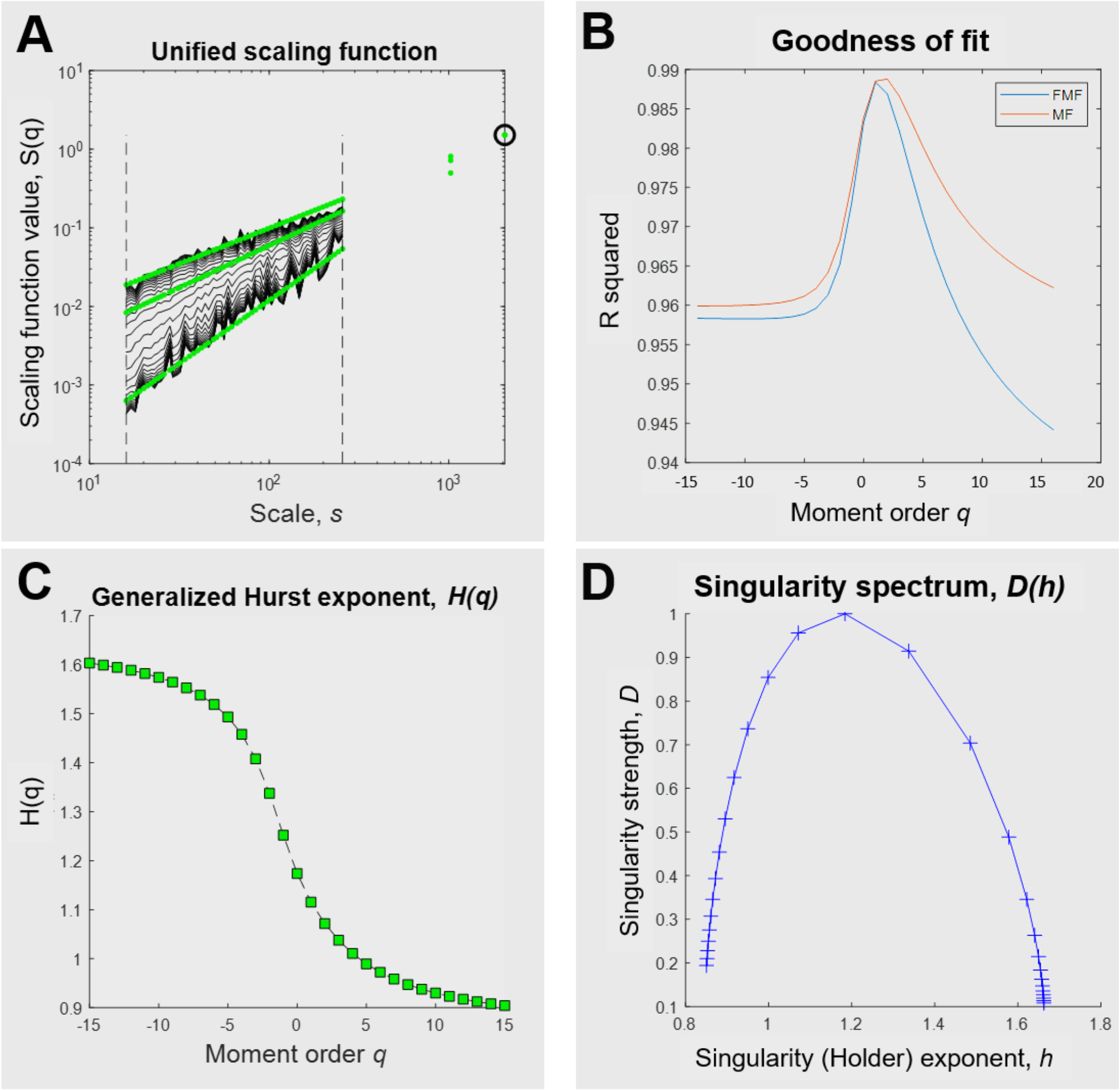

Figure 3 : Multifractal analysis of hand movement time series (in this example, in *UVF* with the dominant hand, same subject as in the figure presented in the main text). (A) Unified scaling function from a focus-based multifractal DFA analysis. (B) Goodness of fit : R^2 of the scaling function for each q value, with (*FMF*, in blue) and without (*MF*, in red) the focus point. (C) Generalized Hurst exponent, obtained from linear regression over statistical moments q. *H(2)* represents the monofractal exponent. The degree of multifractality *ΔH15* is calculated as the difference between H(-15) and H(15). (D) Singularity Spectrum *D(h)*. The width of the singularity spectrum is often used as the multifractal index of the signal.

3 Multifractal properties computed with the Chhabra Jensen method

We conducted a supplementary analysis with the Chhabra Jensen method. Here, the multifractal index is the width of the multifractal spectrum, hereafter labelled *MF-width*.

We removed 5 outliers (detected using the isoutlier function in Matlab). The analysis is therefore conducted on 57 subjects. We chose to conduct the analysis with the same minimum and maximum

scales as the FMF-DFA, FMF-SSC and FMF-WL ones. Therefore, the temporal scales range from 2^4 to 2^8 samples (16 to 256 samples). The range of q used here is determined with different criteria than those employed in above analyses. In contrast to the methods using the focus point, the correlation coefficient R^2 is critical to choose the values resulting from statistical moments q to include in the analysis. Here, we computed the range of q for each subject yielding $R^2 > 0.99$, and kept the minimal range for which each time series passed the cut. This approach led to consider only q values ranging from $q = -1$ to $q = 2$ to establish the multifractal spectrum width, thus multifractality in the time series.

Shapiro–Wilk tests indicated normal distribution of the data in each condition (*LVF, UVF, Standard*) for both hands (*dominant* and *non-dominant*) [all $p > 0.05$].

A two-way ANOVA performed with *condition* and *hand* as independent variables showed an interaction effect [$F(2,341) = 23.39$, $p < 0.05$].

The ANOVA with repeated measurements highlighted differences between *ΔH15* measured on each condition for both hands [$F(5,341) = 10.84$, $p = 1.11 \times 10^{-9}$, $\omega^2 = 0.13$].

Mauchly's Test of Sphericity indicated that the assumption of sphericity was violated [$W = 0.63$, $p = 0.03$]. Sphericity corrections were applied [$p = 3.52 \times 10^{-18}$].

Post-hoc Tuckey tests indicated higher *MF-width* values for the *Standard* condition compared to the *LVF* and *UVF* ones for the dominant hand [all $p < 2 \times 10^{-4}$]. For the non-dominant hand, we found a significatively higher *MF-width* value for the *Standard* compared to the *LVF* condition [$p = 2.35 \times 10^{-6}$], but, unlike previous analysis methods, not between the *Standard* and *UVF* ones [$p = 0.40$].

We found no differences between *LVF* and *UVF* for the dominant hand [$p = 1.00$], whereas there was one for the non-dominant hand [$p = 0.01$]. All the results from post-hoc Tuckey are presented in table 3.

Overall, the results are mainly the same as the ones obtained with the focus-based methods (DFA, SSC and WL). However, unlike the previous results, this method does not find significant differences between the *UVF* and *Standard* conditions for the non-dominant hand.

**Condition 1**	**Condition 2**	**Difference in means**	**p-value**
LVF dominant	UVF dominant	0.00	1.00
LVF dominant	Standard dominant	-0.03	2.82×10^{-4}
LVF dominant	LVF non dominant	0.01	0.96
LVF dominant	UVF non dominant	-0.02	0.13
LVF dominant	Standard non dominant	-0.03	1.68×10^{-4}
UVF dominant	Standard dominant	-0.03	1.82×10^{-8}
UVF dominant	LVF non dominant	0.00	0.97

UVF dominant	UVF non dominant	-0.02	0.10
UVF dominant	Standard non dominant	-0.03	1.07×10^{-4}
Standard dominant	LVF non dominant	0.04	4.36×10^{-6}
Standard dominant	UVF non dominant	0.01	0.48
Standard dominant	Standard non dominant	0.00	1.00
LVF non dominant	UVF non dominant	-0.02	0.01
LVF non dominant	Standard non dominant	-0.04	2.35×10^{-6}
UVF non dominant	Standard non dominant	-0.01	0.40

Table 3 : Results from post-hoc Tuckey for the multifractal width calculated with Chhabra Jensen (q = -1:2, temporal scales 2^4:2^8).

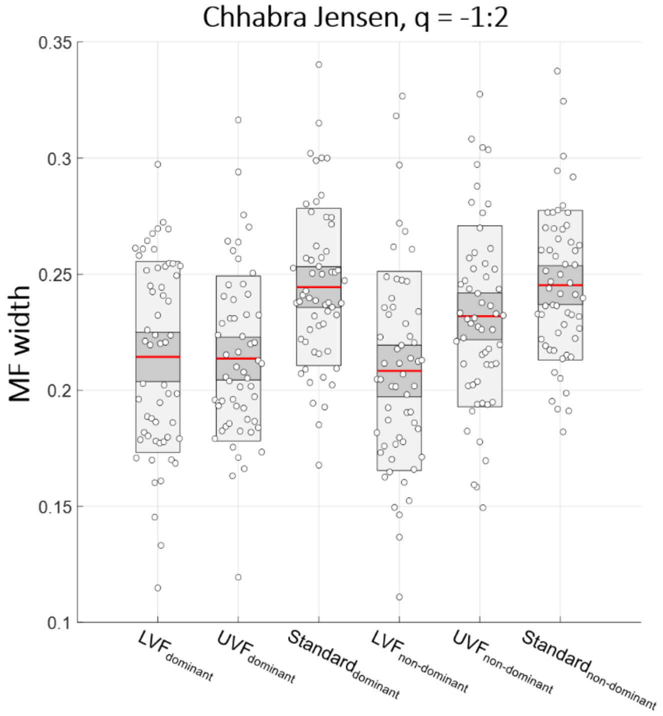

Figure 4 : Multifractal width of the singularity spectrum calculated with the Chhabra Jensen method in the three conditions (*LVF*, *UVF*, *Standard*) for the dominant and non-dominant hands (from left to right), using the maximum shared range of *q* exhibiting $R^2>0.99$ among subject (q = -1:2).
