# Supplementary material for: Fast Hand Movements Unveil Multifractal Roots of Adaptation in the Visuomotor Cognitive System
Source: Front Physiol. 2021 Jul 20;12:713076. doi: 10.3389/fphys.2021.713076 (PMC8330832; doi:10.3389/fphys.2021.713076)
Supplement: Supplementary file 2 [file Table_2.pdf]

Supplementary Material 2

1 Event time series – monofractal properties

In this document, we detail an analysis conducted on the inter-hits time series, *i.e* the times between two successively hit LEDs.

As most of 60s runs in our conditions produced less than 100 samples, especially in the Standard condition, the analyses have been conducted on series created by stitching the inter-hits intervals from both test and retest in each condition.

The monofractal behavior has been estimated using both a DFA-based analysis and the ARFIMA(0,d,0) model (using Whittle approximation of the maximum likelihood estimator).

1.1 MF-DFA

The first calculations were conducted using the *H(2)*, *i.e* the generalized Hurst exponent estimated with *q*=2. The method used here was the same as the one described in the manuscript, with a few noticeable differences. We analysed scales from 4 to N/4 samples, and didn’t used the focus-based approach to avoid biased in the *H(2)* estimation.

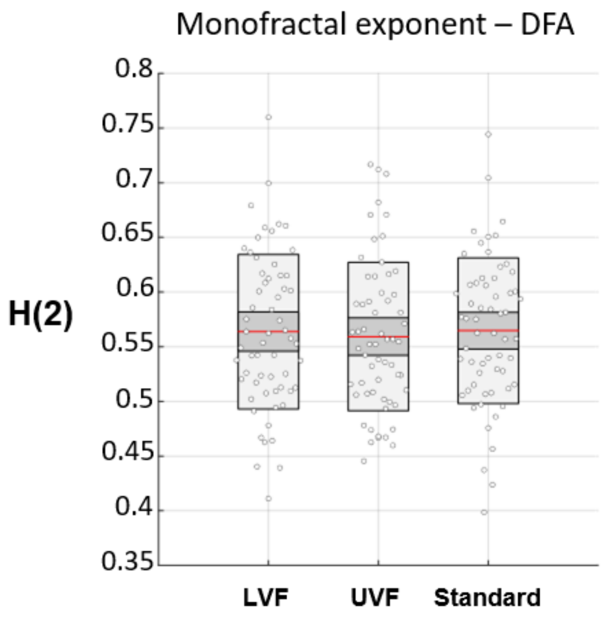

Figure 1 : Monofractal exponent computed with MF-DFA in all conditions

1.2 ARFIMA(0,d,0)

The second method used here derives from the autoregressive fractionally integrated moving average (ARFIMA) model. We used an ARFIMA(0,d,0) model, and estimated d, an indicator of the long range correlations presented in the series. This model only works for fGn (fractional Gaussian noise) series, and provides an estimation of d between]-0.5;0.5[. The monofracal exponent α is then calculated as $\alpha = (2d + 1)/2$.

For series that are not fGn, we differentiated them before ARFIMA computation, and added 1 to the resulting d.

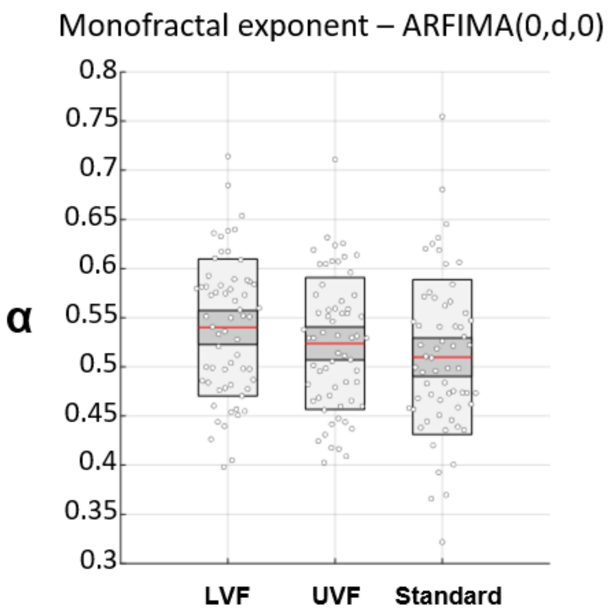

Figure 2 : Monofractal exponent computed with ARFIMA(0,d,0) in all conditions
